# High Concentrations of Rosiglitazone Reduce mRNA and Protein Levels of LRP1 in HepG2 Cells

**DOI:** 10.3389/fphar.2017.00772

**Published:** 2017-11-14

**Authors:** Alejandro N. Rondón-Ortiz, Christian L. Lino Cardenas, Jimena Martínez-Málaga, Ana L. Gonzales-Urday, Kuljeet S. Gugnani, Mark Böhlke, Timothy J. Maher, Alejandro J. Pino-Figueroa

**Affiliations:** ^1^Department of Pharmaceutical Sciences, MCPHS University, Boston, MA, United States; ^2^Cardiovascular Research Center, Massachusetts General Hospital, Boston, MA, United States; ^3^Scientific Consulting Group, BioMolecular-LC E.I.R.L, Arequipa, Peru; ^4^Department of Pharmaceutical, Biochemical and Biotechnological Sciences, Catholic University of Santa Maria, Arequipa, Peru

**Keywords:** LRP1, rosiglitazone, PPARγ, protein degradation, lysosomal degradation, bafilomycin A1

## Abstract

Low-density lipoprotein receptor-related protein 1 (LRP1) is an endocytic receptor involved in the uptake of a variety of molecules, such as apoE, α2-macroglobulin, and the amyloid β peptide (Aβ), for either transcellular transport, protein trafficking or lysosomal degradation. The *LRP1* gene can be transcribed upon activation of peroxisome proliferator receptor activated-γ (PPARγ) by the potent PPARγ agonist, rosiglitazone (RGZ). In previous studies, RGZ was shown to upregulate LRP1 levels in concentrations between 0.1 and 5 μM in HepG2 cells. In this study, we sought to replicate previous studies and to investigate the molecular mechanism by which high concentrations of RGZ reduce LRP1 levels in HepG2 cells. Our data confirmed that transcriptional activation of *LRP1* occurred in response to RGZ at 3 and 10 μM, in agreement with the study reported by Moon et al. (2012a). On the other hand, we found that high concentrations of RGZ decreased both mRNA and protein levels of LRP1. Mechanistically, transcriptional dysregulation of *LRP1* was affected by the downregulation of PPARγ in a time- and concentration-dependent manner. However, downregulation of PPARγ was responsible for only 40% of the LRP1 reduction and thereby the remaining loss of LRP1 (60%) was found to be through degradation in the lysosomal system. In conclusion, our findings demonstrate the mechanisms by which high concentrations of RGZ caused LRP1 levels to be reduced in HepG2 cells. Taken together, this data will be helpful to better explain the pharmacological modulation of this pivotal membrane receptor by PPARγ agonists.

## Introduction

Low-density lipoprotein (LDL) receptor-related protein 1 (LRP1) is a transmembrane receptor that belongs to the LDL receptor family. LRP1 is ubiquitously expressed and has an important role in the transport and uptake of molecules (Lillis et al., 2008; Sagare et al., 2012). Indeed, LRP1 has been involved in the endocytosis of more than 40 structurally different ligands, including apoE, α_2_-macroglobulin, receptor associated protein (RAP) and the amyloid β peptide (Lillis et al., 2008; Ramanathan et al., 2015).

LRP1 consists of two chains that are non-covalently associated. The extracellular α-chain (515 KDa), composed of four ligand-binding clusters, is involved in the recognition and uptake of a diversity of LRP1 ligands. The transmembrane and cytoplasmic β-chain (85 KDa) binds adaptor proteins to facilitate mostly clathrin-mediated endocytosis (Lillis et al., 2008; Kanekiyo et al., 2013) and the more recently demonstrated caveolin-dependent endocytosis (Kanai et al., 2014).

At the transcriptional level, the *LRP1* gene is regulated by PPARγ, due to the presence of the peroxisome proliferator response element (PPRE) on the *LRP1* promoter region (Gauthier et al., 2003). PPARγ is a nuclear receptor involved in important biological processes, including adipogenesis and metabolism. This nuclear receptor is an interesting pharmacological target, since its activation regulates expression of proteins and enzymes involved in glucose metabolism, such as GLUT-2, GLUT-4, IRS-1, IRS-2, PI3K and others (Kim and Ahn, 2004; Ahmadian et al., 2013). Rosiglitazone (RGZ) is a member of the thiazolidinedione family and is a potent PPARγ agonist, known for its antidiabetic properties. However, RGZ has been associated with cardiovascular risks, resulting in a reconsideration of its therapeutic use (Nissen and Wolski, 2007; Hiatt et al., 2013). Indeed, results of some studies have indicated that RGZ modulates LRP1 expression by targeting PPARγ expression in cell culture models, including the HepG2 cell line (Gauthier et al., 2003; Moon et al., 2012a,b).

In this study, we proposed to replicate previous observations and to further explore the effects of RGZ on LRP1, by utilizing HepG2 cells. Our findings confirmed that transcriptional activation of LRP1 is induced by 3 and 10 μM RGZ, whereas higher concentrations (30–100 μM) reduce LRP1 mRNA levels. On the other hand, LRP1 protein levels remained steady in response to 0–10 μM RGZ, but concentrations of RGZ higher than 30 μM dramatically reduced LRP1 protein levels. We subsequently focused our investigation to identify the possible mechanisms by which high concentrations of RGZ decrease LRP1 levels. Indeed, we found that both mRNA and protein levels were negatively affected by two different mechanisms. LRP1 mRNA was reduced by the downregulation of PPARγ in a time- and concentration-dependent manner. Of interest, this mechanism was only responsible for 40% of the reduction of LRP1. The remaining LRP1 protein was found to undergo lysosomal degradation. These results might help to determine whether the side effects caused by RGZ, including those related to cardiovascular risk, are associated with LRP1 reduction.

## Materials and Methods

### Materials

MEM-alpha cell culture medium, OptiMEM reduced-serum medium, fetal bovine serum (FBS), penicillin and streptomycin were obtained from Gibco. Rosiglitazone, T0070907, MG132, bafilomycin A1, chloroquine diphosphate, pepstatin A and E64d were from Tocris. Anti-LRP1 (ab92544); anti-LDLR (ab52818) anti-β-actin (ab49900); secondary antibodies: HRP-conjugated goat anti-rabbit IgG (ab97051), HRP-conjugated goat anti-mouse IgG (ab97023), Alexa Fluor 488 conjugated goat anti-rabbit IgG (ab150077) and Alexa Fluor 594 conjugated goat anti-mouse IgG (ab150116), and concanamycin A were from Abcam. The anti-LC3α/β antibody (SC-398822) was purchased from Santa Cruz Biotechnologies. The anti-LAMP1 (D4O1S) and anti-caveolin-1(D46G3) were purchased from Cell Signaling. 6-Carboxyfluorescein-labeled (FAM) Aβ_1-42_ was obtained from Anaspec. TRIzol reagent and the transcriptor first strand cDNA synthesis kit were from Thermo Fisher and Roche, respectively. DMSO and other reagents were from Sigma–Aldrich.

### Cell Culture

Hepatocellular carcinoma HepG2 cells were obtained from the American Type Culture Collection (HB-8065, ATCC). Cells were seeded in MEM-alpha medium, supplemented with 10% FBS and 1% penicillin/streptomycin. Cells were then grown in a humidified environment with 5% CO_2_ until they were confluent. For experiments, HepG2 cells were seeded into 12-well (2.5 × 10^5^ cells/well) or 6-well (5 × 10^5^ cells/well) plates and were cultured for 48 h, for uptake and protein analysis experiments, respectively. Cells were then exposed to different concentrations of RGZ (3 nM–30 μM) in reduced-serum medium (OptiMEM). The final concentration of DMSO in each experiment was less than 0.5%. Other experiments were carried out in MEM containing 10% FBS, as explained in the Supplementary Information Section.

### RNA Isolation and cDNA Synthesis

Following treatments, RNA was extracted from cells with TRIzol reagent. Isolated RNA was quantified utilizing a NanoDrop 2000 spectrophotometer (Thermo Fisher). cDNA was then prepared with the transcriptor first strand cDNA synthesis kit according to the manufacturer’s protocol. The final cDNA solution was stored at -20°C prior to analysis.

### Quantitative Real-Time PCR (RT-qPCR)

cDNA from samples was analyzed and quantified by RT-qPCR using TaqMan assay probes for *LRP1* (Hs00233856_m1), *PPARG* (Hs00234592_m1) and *ACTB* (Hs01060665_g1) in a StepOne Plus station (Applied Biosystems). This reaction was carried out in triplicate in microamp optical 96-well plates in a total volume of 10 μL/well. The relative *LRP1* expression levels were calculated as the fold-change determined by the ΔΔCt method with *ACTB* representing a housekeeping gene.

### Cell Viability

HepG2 cells were seeded into 96-well plates and were exposed to different concentrations of RGZ. Treatments were carried out with the control group consisting of DMSO-treated cells. Cell viability was determined by the colorimetric method utilizing 3-(4,5-dimethylthiazol-2-yl)-5-(3-carboxymethoxy-phenyl)-2-(4-sulfophenyl)-2H-tetrazolium salt (MTS, CellTiter 96^®^AQueous One Solution, Promega). Cell viability was determined at 490 nm and was calculated as percent of control.

### Protein Extraction and Western Blot

HepG2 cells were rinsed twice with ice-cold PBS and proteins were extracted with M-PER and MEM-PER, for whole cell lysis and membrane isolation, respectively (both from Thermo Fisher). These lysis buffers contained Halt protease, phosphatase inhibitors and EDTA (Thermo Fisher). The protein concentration was determined by the colorimetric bicinchoninic acid assay (BCA assay, Thermo Fisher). Equal amounts of total protein from cell lysates were separated by SDS-PAGE (20 and 40 μg for LRP1 and LC3, respectively). Proteins from the gel were then electro-transferred onto 0.45 μm nitrocellulose and 0.2 μm PVDF membranes for LRP1 and LC3, respectively.

The membranes were then blocked for 1 h at room temperature (rt), with either 5% non-fat powdered milk dissolved in TBS-T or 5% bovine serum albumin in TBS-T, for the nitrocellulose and PVDF membranes, respectively. Following blocking, membranes were incubated overnight at 4°C with the primary antibodies anti-LRP1 (1:10,000), anti-LDLR (1:2,000), anti-caveolin1 (1:1,000) and anti-LC3 (1:500). The membranes were subsequently washed three times with TBS-T and were then exposed to HRP-conjugated goat anti-rabbit IgG (1:2,000) or HRP-conjugated goat anti-mouse IgG (1:2,000) for 1 h at rt. β-Actin was used as the loading control; the membranes were incubated with HRP-conjugated mouse polyclonal anti-β-actin (1:10,000) for 1 h at rt. SuperSignal West Pico chemiluminescent substrate (Thermo Fisher) was added to the membranes and they were incubated for 5 min. Bands were visualized with the C-DiGit blot scanner (Licor Technologies).

### Cell Transfection

HepG2 cells were grown in MEM alpha medium. The LRP1 construct (Mini LRP-IV-EGFP) was kindly provided by Dr. Michel Khrestchatisky and Dr. Marion David (Université d’Aix-Marseille and Vect-Horus, Marseille, France, respectively; Perrot et al., 2012). Cells were transiently transfected with 1.5 μg of pDNA using Lipofectamine 2000 (Thermo Fisher), following manufacturer’s protocol.

### Confocal Microscopy

For immunocytochemistry, HepG2 cells were cultured into 8-well Lab-Tek^TM^ II Chamber Slides (Nunc^TM^) and were then treated with either RGZ or inhibitors. Cells were rinsed twice with ice-cold PBS, fixed with 4% paraformaldehyde in PBS (PFA, Boston Bioproducts) for 15 min at rt, and were permeabilized with 0.1% Triton-X (Sigma–Aldrich) for 10 min. The slides were blocked with 10% goat-serum, and 0.3 M glycine in PBS-Tween 20 (0.1%) for 1 h at rt. Subsequently, the antibodies anti-LRP1 (1:100), anti-LAMP1 (1:50) and anti-LC3 (1:50) were added and slides were incubated overnight at 4°C. The slides were then washed 3 times for 5 min each with PBS-T and were incubated with Alexa Fluor 488 conjugated goat anti-rabbit IgG and Alexa Fluor 594 conjugated goat anti-mouse IgG (each at 1:400 dilution) for 1 h at rt. Following immunostaining, slides were mounted with diamond mounting medium containing DAPI (Thermo Fisher). Slides were then visualized with the Leica TCS SP8 confocal microscopy station and the pictures were digitized with the Leica Application Suite X software. Recordings of the 3D-reconstruction can be found in the Supplementary Information Section. For time-lapse imaging microscopy, HepG2 cells were cultured in 35 mm μ-Dishes (Ibidi) overnight. Following transfection with the mini-LRP-IV-EGFP plasmid, cells were treated with either vehicle or RGZ (30 μM) for 22 h. Deep red lysotracker (Thermo Fisher) was added to visualize the lysosomal compartment. Single z-plane images were recorded every 3 min for the next 2 h from both control and RGZ-treated cells. Cells were visualized with the Leica TCS SP8 confocal microscopy station and pictures were digitized with the Leica Application Suite X software. Recordings of time-lapse imaging can be found in the Supplementary Information Section.

### Aβ_1-42_ Preparation and Uptake

Synthetic FAM-Aβ_1-42_ peptide was resuspended as previously described (Fuentealba et al., 2010; Kanekiyo et al., 2013). Briefly, FAM-Aβ_1-42_ was resuspended in 1,1,1,3,3,3-hexafluoroisopropanol and aliquoted into amber tubes. The solvent was evaporated with a stream of nitrogen gas and tubes containing the dried film were stored at -20°C until they were utilized for uptake studies. For experiments, the films were resuspended in DMSO to obtain a 100 μM stock solution and were then diluted to 500 nM FAM-Aβ_1-42_ in OptiMEM. HepG2 cells were treated with RGZ (30 μM) for 24 or 48 h. Following this treatment, HepG2 cells were incubated with fresh FAM-Aβ_1-42_ (500 nM) for 30 min, 1 and 2 h. Cells were then lysed with M-PER, as above, and fluorescence intensity was monitored with the Synergy HT plate reader (Biotek), with excitation at 485/20 nm and emission at 528/20 nm. FAM-Aβ_1-42_ uptake is expressed as relative fluorescence units per mg protein (RFU/mg).

### Statistical Analysis

All statistical analyses were conducted using GraphPad Prism 6. Data are reported as mean ± SEM for at least three independent experiments. Comparisons between two or multiple groups were performed by a two-tailed Student’s *t*-test and one-way analysis of variance (ANOVA), respectively. The ANOVA was followed by a *post hoc* Dunnet’s test, in which experimental groups were compared to controls (cells treated with vehicle). A value of *p* < 0.05 was considered to demonstrate a significant difference between treatments.

## Results

### Effect of RGZ on LRP1 Levels

**FIGURE 1:**
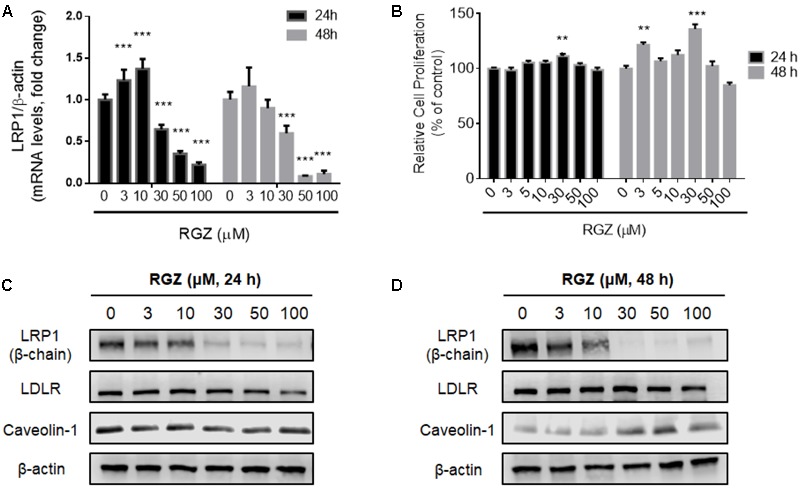
Effects of RGZ on LRP1 levels in HepG2 cells. RGZ (3 and 10 μM) increased LRP1 mRNA levels at 24 h. However, the highest concentration of RGZ (30 μM) reduced mRNA levels significantly and this effect was also observed at 48 h (**A**, *n* = 6). Cell proliferation assay demonstrated that RGZ did not cause cytotoxicity at these concentrations (**B**, *n* = 6). LRP1 protein levels remained steady (β-chain 85 Kda) in response to 3 and 10 μM RGZ compared to control for 24 and 48 h. Nonetheless, concentrations higher than 10 μM RGZ reduced LRP1 protein levels (**C,D**, representative blots). Data are presented as mean ± SEM. In the one-way ANOVA, followed by Dunnet’s test and compared to control group, *p* < 0.05 was considered significant (^∗^*p* < 0.05; ^∗∗^*p* < 0.01; ^∗∗∗^*p* < 0.001).

Previous studies demonstrated the modulation of LRP1 by the transcription factor PPARγ in cells treated with RGZ (Gauthier et al., 2003; Moon et al., 2012a). In order to confirm the RGZ effect on LRP1 expression, we replicated the experiments previously reported, which utilized HepG2 cells (Moon et al., 2012a). In agreement with the literature, we observed the transcriptional activation of LRP1 mRNA in cells treated with RGZ at 3 and 10 μM for 24 h (**Figure 1A**). However, we did not observe increases in LRP1 protein levels following either 24 or 48 h exposures to these concentrations of RGZ (**Figures 1C,D**). Other variables were also tested, such as incubation time, RGZ concentration and presence of serum in the treatments, but we did not observe the LRP1 protein upregulation previously described (Moon et al., 2012a, **Figure 1** and Supplementary Figure 1). In contrast, higher concentrations than 10 μM RGZ decreased both mRNA and protein LRP1 levels significantly after 24 and 48 h treatments. In addition, the effect of RGZ seemed to be specific to reduce LRP1, since LDLR (another member of the LDL receptor family that undergoes clathrin-dependent endocytosis) and caveolin-1 (a marker for caveolae-dependent endocytosis) were not altered in the presence of RGZ (**Figures 1C,D**). We established that these effects were not due to cytotoxicity, as cell viability was maintained at these RGZ concentrations (**Figure 1B** and Supplementary Figure 1).

### High Concentration Rosiglitazone Reduces both LRP1 mRNA and Protein Levels

**FIGURE 2:**
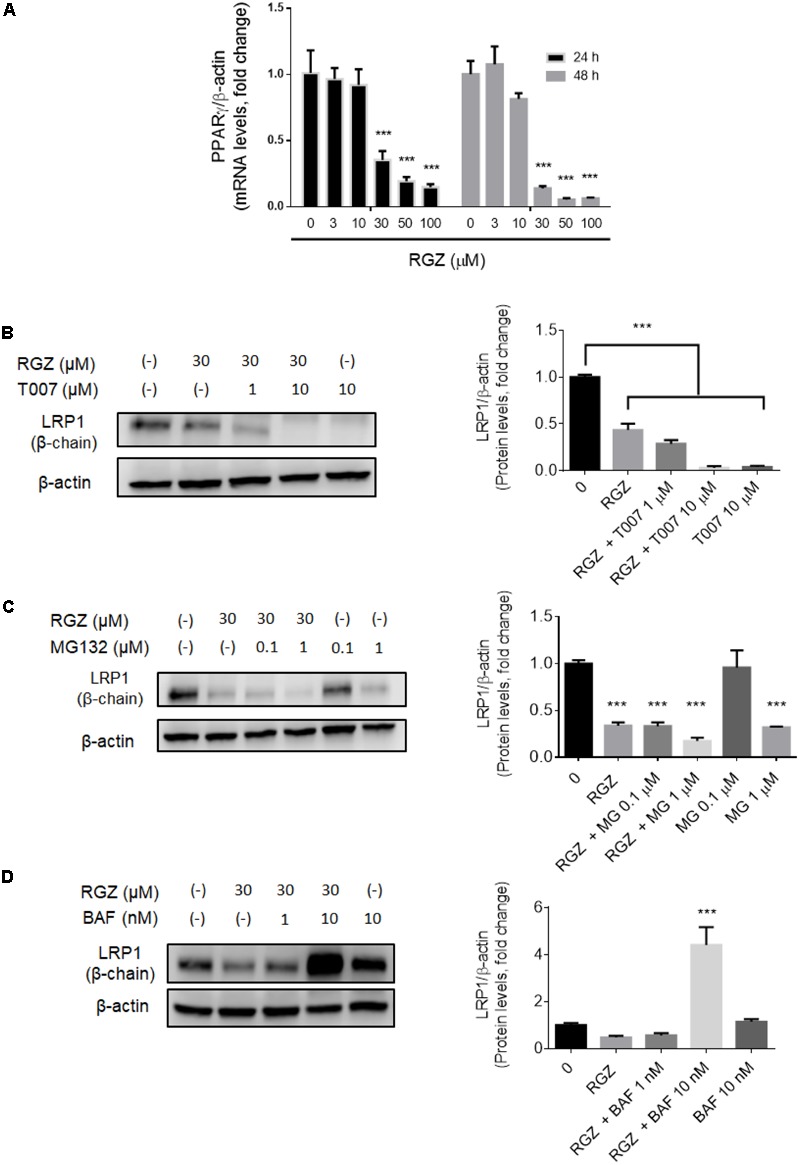
High concentrations of RGZ reduced both LRP1 mRNA and protein by two independent mechanisms in HepG2 cells. LRP1 mRNA reduction might be a consequence of PPARγ downregulation induced by RGZ in a time-and concentration-dependent manner (**A**, *n* = 6). This process is confirmed when PPARγ is antagonized by T007. T007 reduced LRP1 protein levels in a concentration-dependent manner (**B**, *n* = 4). UPS inhibition by MG132 did not prevent LRP1 degradation (**C**, *n* = 4). However, lysosomal degradation inhibitor (Bafilomycin A1, BAF) increased LRP1 by up to a four-fold change in RGZ-treated HepG2 cells compared to control group after 24 h incubation (**D**, *n* = 4). Data are presented as mean ± SEM. In the one-way ANOVA, followed by Dunnet’s test and compared to the control group, *p* < 0.05 was considered significant (^∗^*p* < 0.05; ^∗∗^*p* < 0.01; ^∗∗∗^*p* < 0.001).

The *LRP1* gene contains the PPRE that is recognized by activated PPARγ to begin the transcription of *LRP1* (Gauthier et al., 2003; Moon et al., 2012a,b; Kim et al., 2014). However, we observed that higher concentrations than 10 μM RGZ reduced LRP1 mRNA levels in a time- and concentration-dependent manner (**Figure 1A**). Previous studies demonstrated that high concentrations of PPARγ agonists, including RGZ and pioglitazone, reduce PPARγ levels (Hauser et al., 2000; Kilroy et al., 2009). We observed that LRP1 mRNA reduction is a consequence of the PPARγ downregulation resulting from exposures of cells to RGZ at concentrations higher than 10 μM in a time- and concentration-dependent manner, as shown in **Figure 2A**. In addition, we tested whether PPARγ antagonism by T0070907 (T007) affects LRP1 protein levels. Our observation was that PPARγ antagonism reduced LRP1 protein levels in a concentration-dependent manner (**Figure 2B**).

While reduced LRP1 mRNA levels can be explained by PPARγ downregulation, we observed that high concentrations of RGZ reduced LRP1 protein levels to a greater extent than *LRP1* transcription (**Figure 1**). This suggests that in addition to the transcriptional effect on LRP1 levels, a possible proteolytic mechanism might be affecting LRP1 protein levels in RGZ-treated HepG2 cells. We then investigated two proteolytic degradation mechanisms as possible explanations for these observations: the ubiquitin proteasome system (UPS) and lysosomal degradation.

The ubiquitin proteasome system (UPS) is the primary mechanism for protein degradation in eukaryotic cells (Vilchez et al., 2014). Previous studies have demonstrated that the pharmacological activation of PPARγ promotes its own proteolytic degradation via UPS (Hauser et al., 2000; Kilroy et al., 2009). In addition, it has been proposed that UPS is the key regulator for LRP1 degradation in a number of cell lines, including HepG2 (Melman et al., 2002; Cal et al., 2013). The use of MG132, a potent UPS inhibitor, in HepG2 cells treated with RGZ (30 μM) for 24 h did not prevent LRP1 protein degradation, as shown in **Figure 2C**. We also observed that MG132 reduced LRP1 protein levels in a concentration-dependent manner (**Figure 2C** and Supplementary Figure 2). These results excluded the possibility that LRP1 degradation occurred by the UPS pathway in RGZ-treated HepG2 cells. This suggested that RGZ reduced the amount of LRP1 present by a mechanism other than UPS.

Since LRP1 has been demonstrated to be involved in the transport of its ligands to the lysosomal compartment for degradation (Laatsch et al., 2012; Gan et al., 2014; Zhao et al., 2015), we decided to study the status of lysosome proteolytic degradation in RGZ-treated HepG2 cells. Cells were incubated with bafilomycin A1 (BAF), a vacuolar H^+^-ATPase inhibitor which blocks lysosomal activity, in the presence or absence of RGZ (30 μM) for 24 h. Western blot analysis demonstrated increased LRP1 protein levels, up to a 4-fold change, in cells co-treated with BAF (10 nM) and RGZ (30 μM) compared to the control, as depicted in **Figure 2D**. These results suggest that during RGZ treatment, LRP1 undergoes proteolytic degradation through the lysosomal system.

### High Concentration RGZ Promotes LRP1 Lysosomal Degradation in HepG2 Cells

**FIGURE 3:**
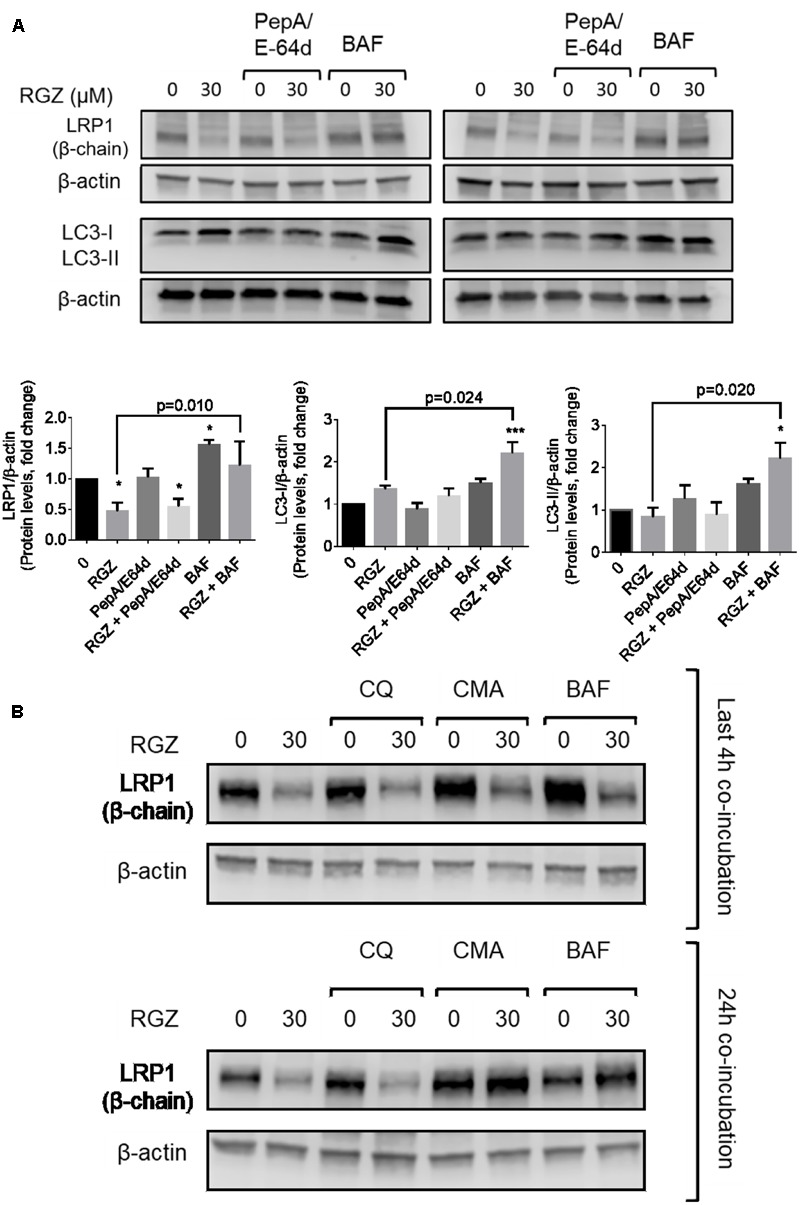
LRP1 protein levels are restored by lysosomal activity inhibitors in RGZ-treated HepG2 cells. BAF prevented LRP1 degradation in the last 4 h of exposure, whereas cathepsin inhibitors (PepA/E64d) failed to prevent LRP1 degradation. RGZ is associated with induced autophagy in cancer cells, as observed in the increase of LC3-II when lysosomal activity is blocked by BAF (**A**, *n* = 4). The lysosomotopic agent chloroquine (CQ) failed to prevent LRP1 degradation, while the potent vacuolar-type H^+^ ATPase inhibitor concanamycin A (CMA) prevented RGZ-induced LRP1 degradation (**B**, representative blot). Data are presented as mean ± SEM. In the one-way ANOVA, followed by Dunnet’s test and compared to the control group, *p* < 0.05 was considered significant (^∗^*p* < 0.05; ^∗∗^*p* < 0.01; ^∗∗∗^*p* < 0.001). Then, a two-tailed Student’s *t*-test was performed to compare RGZ vs. RGZ and BAF and a *p* < 0.05 was considered significant.

**FIGURE 4:**
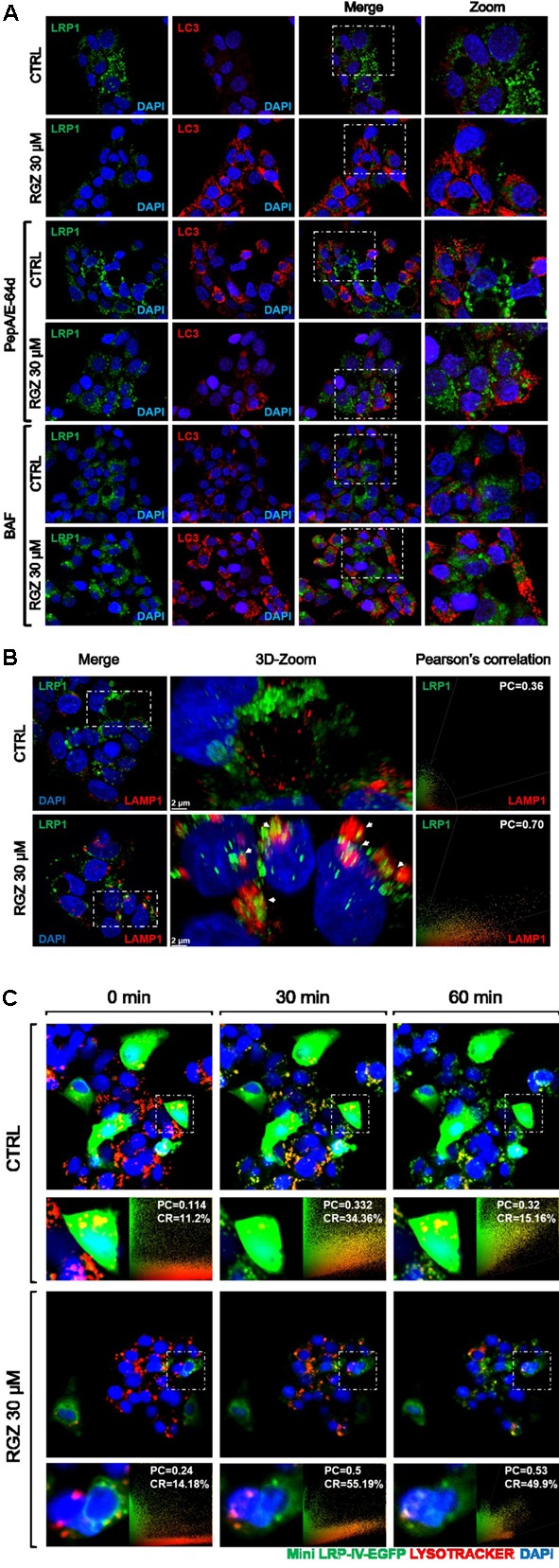
RGZ promotes LRP1 lysosomal degradation independently of autophagy. RGZ increased LC3 puncta in HepG2 cells, whereas LC3 did not co-localize with LRP1. This suggests that LRP1 is degraded by a process other than autophagy (**A**, representative slide). RGZ promoted LRP1/LAMP1 co-localization demonstrated by 3D-reconstruction confocal microscopy to a higher extent (PC = 0.70), compared with control (PC = 0.36); as observed in **(B)** (a representative z-projection one of 13 to 16 cuts). Deep red lysotracker was incubated with ectopically over-expressed fluorescent LRP1 construct (Mini-LRP-IV-EGFP) HepG2 cells after 22 h of RGZ treatment. Time-lapse imaging was used to monitor both lysotracker and GFP signal. Mini-LRP-IV-EGFP co-localized with positive lysosome compartment sensor and GFP signal was reduced in RGZ-treated cells compared with control, suggesting that RGZ promoted lysosomal degradation of ectopically over-expressed LRP1 **(C)**. PC: Pearson’s co-localization, CR: Co-localization rate.

A long exposure of RGZ-treated HepG2 cells to BAF might affect other non-specific cellular events, such as UPS activation and protein aggregation, as previously reported (Korolchuk et al., 2009; Rubinsztein et al., 2009). We reduced BAF incubation time to the last 4 h of the experiment (total time of 24 h). In addition, cells were exposed to a mixture of the cathepsin inhibitors pepstatin A (PepA) and E64d, each at 30 μM. The purpose of the PepA/E64d mixture was to reduce lysosomal activity. As expected, RGZ reduced LRP1 levels, determined both by immunocytochemistry and Western blot analysis. On one hand, BAF treatment significantly increased LRP1 signal both in the presence or absence of RGZ, but PepA/E64d did not prevent LRP1 degradation, probably because PepA and E64d act downstream from BAF (**Figures 3A, 4A**). This suggests that BAF is capable to prevent the lysosomal degradation of LRP1 caused by RGZ.

BAF inhibits the last stage of autophagy, consisting of lysosomal degradation (Klionsky et al., 2016); and RGZ has been associated with autophagy in cancer cell lines (Zhou et al., 2009; Vara et al., 2013). To assess whether autophagy induced by RGZ is responsible for LRP1 degradation, the autophagosome marker LC3 was analyzed by immunocytochemistry and Western blot. Our observations demonstrated that RGZ (30 μM) induced autophagy by the presence of LC3 puncta, monitored by immunocytochemistry and LC3-II generation by Western blot. Moreover, the presence of BAF induced LC3 accumulation (**Figures 3A, 4A**). In addition, reduced levels of LRP1 were also observed in EBSS-starved HepG2 cells, but BAF prevented LRP1 degradation (Supplementary Figure 3). LRP1 did not co-localize with LC3, suggesting that LRP1 degradation induced by RGZ is not an autophagy-dependent process, but RGZ might promote LRP1 lysosomal degradation (**Figure 4A**).

We found that BAF prevented LRP1 proteolytic degradation induced by RGZ (**Figures 2D, 3A**). We then tested whether other lysosomal activity inhibitors would prevent LRP1 degradation in 30 μM RGZ-treated cells. Our observations were that 10 nM concanamycin A (CMA), another vacuolar H^+^-ATPase inhibitor, had an effect similar to that of BAF in RGZ-treated cells. On the other hand, the lysosomotropic agent chloroquine (CQ, known to increase the lysosomal pH and thus reduce activity of some lysosome proteolytic enzymes) at 30 μM did not prevent RGZ-induced LRP1 degradation (**Figure 3B**). These observations suggest that lysosomal enzymes which are affected by CQ and PepA/E64d, are not responsible for LRP1 degradation. Taken together these results suggest that BAF and CMA, which act more upstream than CQ and PepA/E64d, prevented LRP1 lysosomal degradation caused by 30 μM RGZ.

### High Concentration RGZ Promotes Intracellular Localization of LRP1 with Lysosomes

Since previous observations suggested that 30 μM RGZ might promote LRP1 lysosomal protein degradation (**Figures 2D, 3B**), we immunostained LRP1 and the lysosomal marker LAMP1 to determine LRP1 lysosomal localization. Our findings confirmed that LRP1 co-localizes with LAMP1 in the 3D-reconstruction we obtained using confocal microscopy (**Figure 4B** and Supplementary Figure 4). In addition, HepG2 cells were transfected with a functional LRP1 construct (Mini LRP-IV-EGFP; Perrot et al., 2012). Transfected HepG2 cells were then treated with 30 μM of RGZ for 22 h and we performed time-lapse imaging to monitor this fluorescent construct in the presence of lysotracker over a 1 h period. Lysotracker is a fluorescent dye used to label and to monitor acidic cellular compartments, including lysosomes. Our findings demonstrated that the mini-LRP-IV-EGFP construct co-localized with lysotracker to a greater extent in RGZ-treated cells than in the control. Moreover, the fluorescence intensity was constant in the control, while RGZ-treated cells displayed a reduced fluorescence intensity (**Figure 4C**). This suggested that RGZ induces lysosomal degradation of both endogenous LRP1 protein and ectopically overexpressed fluorescent Mini-LRP-IV-EGFP.

### High Concentration RGZ Reduces LRP1 Activity

**FIGURE 5:**
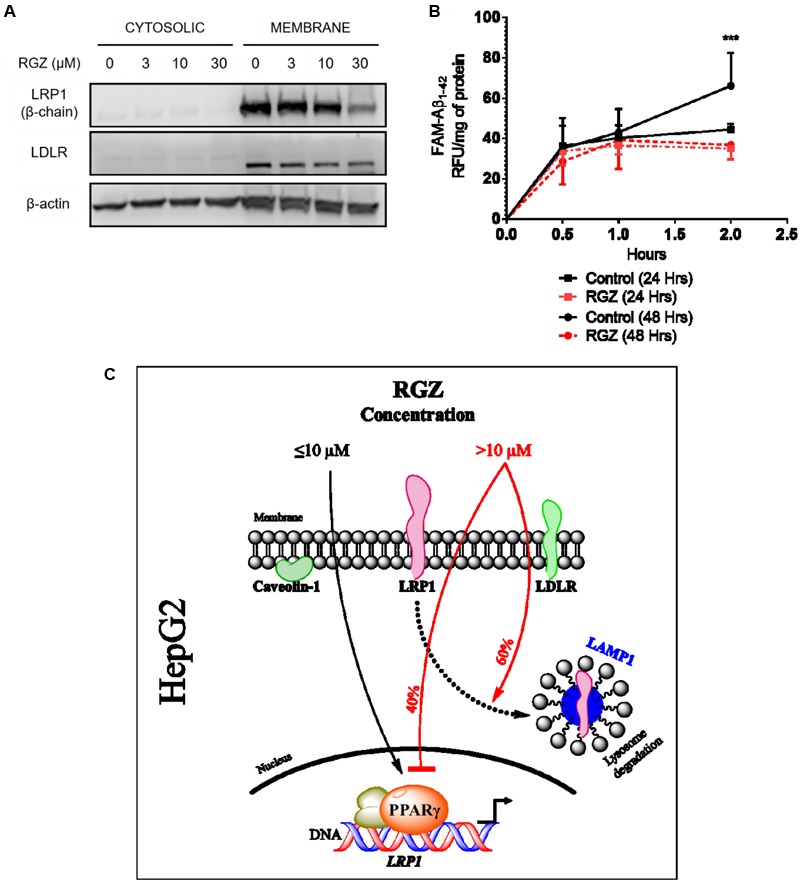
RGZ reduces LRP1 PM levels and impairs LRP1 activity. LRP1 at the cell surface area was significantly reduced by 30 μM RGZ, whereas LDLR PM levels remained steady in response to different concentrations of RGZ (**A**, representative blot). RGZ (30 μM), after 48 h of treatment, impaired FAM-Aβ1-42 peptide uptake during 2 h of incubation. This suggests that RGZ reduced LRP1 activity (**B**, *n* = 6). Summary representation of our study **(C)**. Data are presented as mean ± SEM. A two-tailed Student’s *t*-test was performed to compare RGZ vs. CTRL at 2 h post-incubation with FAM-Aβ1-42 and a *p* < 0.05 was considered significant. (^∗∗∗^*p* < 0.001).

RGZ (30 μM) was demonstrated to reduce both LRP1 mRNA and protein levels, by the mechanisms of downregulation of PPARγ and protein lysosomal degradation, respectively (**Figures 1–3**). Given that the primary role of LRP1 is to endocytose its ligands at the cell surface (Perrot et al., 2012; Kanekiyo et al., 2013; Ramanathan et al., 2015) we evaluated LRP1 plasma membrane (PM) levels in cells treated with different concentrations of RGZ. Our results demonstrate that 30 μM RGZ reduced LRP1 PM levels, whereas 3 and 10 μM did not affect LRP1 PM levels. In addition, LDLR PM levels were not affected by RGZ, suggesting the specificity of RGZ to regulate LRP1 levels (**Figure 5A**).

The fluorescent peptide Aβ_1-42_ (FAM-Aβ_1-42_) is one of the hallmarks of Alzheimer’s disease patients and this peptide was previously demonstrated to have a high affinity for LRP1 (Deane et al., 2004; Kanekiyo et al., 2013) Moreover, hepatic cells have an important role in the clearance and uptake of systemic Aβ via LRP1 endocytosis (Tamaki et al., 2006, 2007; Sagare et al., 2011). Thus, we tested whether the LRP1 reduction caused by 30 μM RGZ affected FAM-Aβ_1-42_ uptake in HepG2 cells. Our findings demonstrated that following 24 and 48 h incubations with RGZ, only the 2 h exposure of FAM-Aβ_1-42_, following the 48 h incubation, resulted in a significant difference in FAM-Aβ_1-42_ uptake compared to control (*p* < 0.001; **Figure 5B**). This impaired uptake correlated with lower LRP1 levels in the whole cell lysates and PM, presented in **Figures 1, 2**, and **5A**.

## Discussion

LRP1 is a ubiquitous transmembrane receptor, with a role in the internalization of more than 40 structurally different ligands, which are either transported to lysosomes for degradation or are transported transcellularly via endosomal trafficking (Sagare et al., 2012; Ramanathan et al., 2015). Previous reports have identified a conserved PPRE consensus sequence on the promoter region of the *LRP1* gene, which can be transcriptionally activated by PPARγ agonists such as rosiglitazone (RGZ) in HepG2, HBMEC and SW872 cells (Gauthier et al., 2003; Moon et al., 2012a,b). A novel molecular interplay between LRP1 and PPARγ has recently been documented, suggesting that LRP1 might act as a co-activator of PPARγ to regulate glucose and lipid homeostasis in endothelial cells (Mao et al., 2017). In addition, LRP1 has been suggested to be involved in the metabolism and uptake of glucose, by interacting with insulin receptor-β (Jedrychowski et al., 2010; Liu et al., 2015). LRP1 PM residency is influenced by insulin signaling, as it has been observed that insulin-stimulated cells have increased LRP1 endocytic activity (Tamaki et al., 2007; Laatsch et al., 2009). Based on this evidence and on the role of LRP1 in cargo transport by endocytosis, there is no doubt that LRP1 dysregulation will affect important cell processes, and could be associated with disease states including diabetes, considering its effects on insulin and glucose metabolism. Moreover, low levels of LRP1 have been observed in Alzheimer’s disease patients (Zhao et al., 2015), in certain cancers (Perrot et al., 2012; Van Gool et al., 2015), in cases of reduced glucose uptake and metabolism (Tamaki et al., 2007; Jedrychowski et al., 2010; Liu et al., 2015) and in certain cardiovascular pathologies (Bown et al., 2011; Wild et al., 2012; Strickland et al., 2014).

RGZ is a synthetic PPARγ agonist, which has been used as an insulin sensitizer in Type 2 diabetes. Other beneficial properties have been attributed to RGZ; it is a renal protective agent, it is a cytoprotective agent and it attenuates the cognitive defects associated with Alzheimer’s disease (Pedersen et al., 2006; Risner et al., 2006; Morrison et al., 2017). However, cardiovascular risks have been associated with RGZ, resulting in a reconsideration of its therapeutic use (Nissen and Wolski, 2007; Hiatt et al., 2013). Of interest, RGZ seems to affect LRP1 levels in a concentration-dependent manner. Low concentrations of RGZ (0–5 μM) have been demonstrated to upregulate LRP1 expression at both the mRNA and protein levels, likely via PPARγ activation (Moon et al., 2012a). However, higher concentrations of RGZ (30–100 μM) seem to have the opposite effect on LRP1 expression. The mechanism by which these concentrations of RGZ reduce LRP1 levels is unclear. In this study, our goal was first to replicate previous observations; and second, to uncover the molecular mechanism by which RGZ at high concentrations reduces both LRP1 mRNA and protein levels.

We have confirmed that at 3 and 10 μM, RGZ activates LRP1 mRNA expression, as previously reported (Gauthier et al., 2003; Moon et al., 2012a). Higher concentrations than 10 μM reduce LRP1 mRNA levels, as a result of PPARγ downregulation, as shown in **Figures 1** and **2**. Previous studies reported that proteolytic degradation of PPARγ occurs via UPS in the presence of high concentrations of RGZ, and that degradation is prevented by the UPS inhibitors: MG132 and lactacystin (Hauser et al., 2000; Kilroy et al., 2009). Our observations suggest that in addition to PPARγ protein degradation via UPS, a transcriptional *PPARγ* downregulation takes place in cells treated with a high concentration of RGZ; as a consequence, PPARγ target genes are compromised.

High concentrations of PPARγ agonists, including RGZ, have been implicated to induce lysosomal degradation processes in cancer cells, for example autophagy (Zhou et al., 2009; Cerquetti et al., 2011). In addition, this statement corroborates the finding that PPARγ downregulation induced autophagy in hepatocellular carcinoma (Vara et al., 2013). Our results are consistent with others in the literature, since high concentrations of RGZ downregulated PPARγ, induced autophagy and promoted LRP1 lysosomal degradation (**Figures 2–4, 5C**).

This study demonstrates that RGZ reduced LRP1 levels at higher concentrations (>10 μM). These concentrations are higher than the maximum plasma concentration (Cmax) observed in healthy patients (652 ng/mL or 1.8 μM) following a dose of 8 mg/kg (Kirchheiner et al., 2006). However, the pharmacokinetic profile of this drug can be altered by affecting its metabolism. RGZ is metabolized primarily by CYP2C8 and to a lesser extent by CYP2C9 (Kirchheiner et al., 2006; Akhlaghi et al., 2017). CYP2C8 inhibition by gemfibrozil increased both RGZ Cmax by 22% and half-life from 3.6 h to 7.6 h (Niemi et al., 2003; Tornio et al., 2012). The CYP2C8 enzyme is genetically polymorphic and the variant CYP2C8^∗^3 encodes a protein with 2 linked amino acid substitutions, Arg139Lys (416G > A) and Lys399Arg (1196A > G). This variant has been reported to be present in 13–15% of the Caucasian population and it has considerably reduced oxidative capacity (Dai et al., 2001). However, the CYP2C8^∗^3 variant conferred a higher activity than wild-type CYP2C8 on RGZ metabolism (Kirchheiner et al., 2006). On the other hand, another genetic variant of CYP2C8 has affected metabolism and exacerbated cerivastatin side effects (Ishikawa et al., 2004). Thus, this study might be helpful to interpret the genetic status of RGZ’s metabolic enzymes of patients who have been medicated with RGZ. In this context, future studies might be required to determine the association of LRP1 reduction and the side-effects observed in RGZ-prescribed patients, including the cardiovascular risk-related effects.

## Conclusion

The results of this study provide new insights into how LRP1 levels are affected by higher concentrations of RGZ. Our findings demonstrate that LRP1 reduction occurs by the mechanisms of both PPARγ downregulation and lysosomal degradation, responses to exposures to high concentrations of RGZ. LRP1 reduction might be associated with some adverse side effects observed in RGZ-prescribed patients.

## Author Contributions

Participated in the experimental design and data analysis: AR-O, CLC, and AP-F. Conducted the experiments: AR-O and CLC, with help of JM-M, AG-U, and KG. Contributed with new reagents and analytical tools: MB, TM, and AP-F. Wrote the first draft of the manuscript: AR-O. Contributed to the writing of the manuscript: AR-O, CLC, MB, TM, and AP-F. All authors read and approved the final version of the manuscript.

## Conflict of Interest Statement

The authors declare that the research was conducted in the absence of any commercial or financial relationships that could be construed as a potential conflict of interest.
