# Supplementary material for: High Concentrations of Rosiglitazone Reduce mRNA and Protein Levels of LRP1 in HepG2 Cells
Source: Front Pharmacol. 2017 Nov 14;8:772. doi: 10.3389/fphar.2017.00772 (PMC5696635; doi:10.3389/fphar.2017.00772)
Supplement: Supplementary file 1 [file Data_Sheet_1.docx]

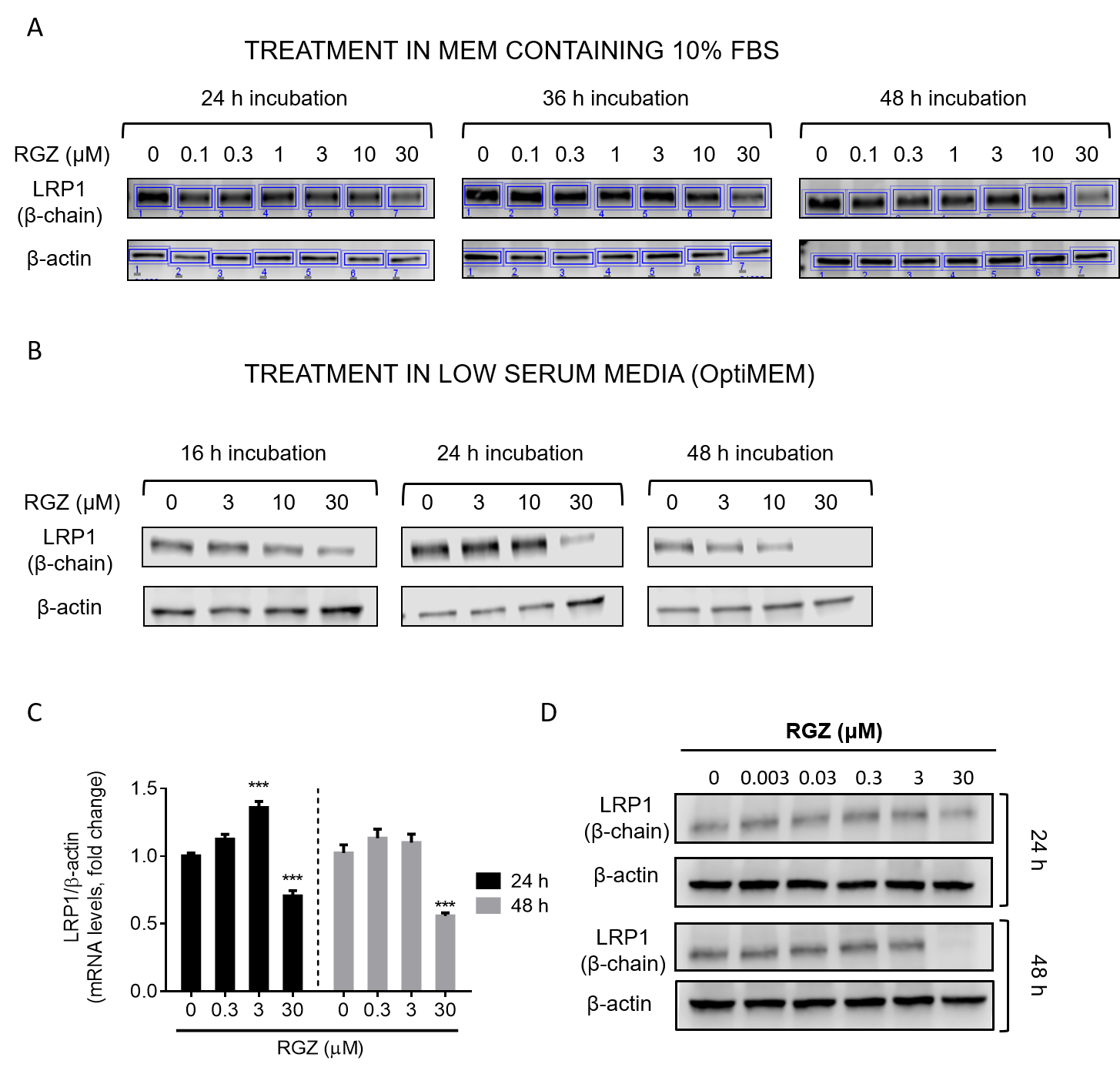


**Supplementary Figure 1.** Representative Western blots of LRP1 protein levels evaluated in this study. Several variables were assessed to reproduce the previous study (Moon, et al, 2012a). In the upper panel, HepG2 cells were treated with different concentrations of RGZ in complete media (MEM-alpha containing 10% FBS, A). Cells were treated with different concentrations of RGZ in reduced serum media (OptiMEM, B - D). Multiple time points were used to extract protein levels in both panels. Different concentrations of RGZ were used to demonstrate the lack of cytotoxicity at high concentrations for 24 h (C) and 48 h (D). Data are presented as mean ± S.E.M. (n=5).


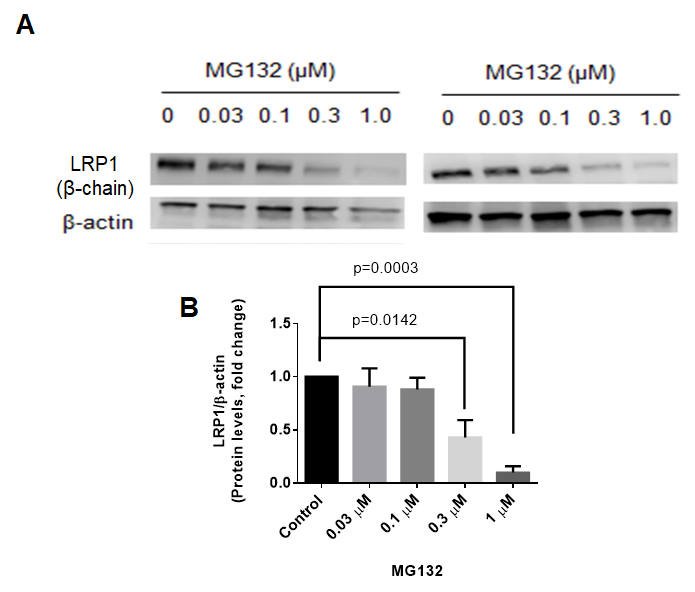


**Supplementary Figure 2.** MG132 reduces LRP1 protein levels in a concentration-dependent manner in HepG2 cells after 24 h incubation. Representative Western blots are displayed in (A) and their densitometric analyses are displayed in (B, n=4). Data are presented as mean ± S.E.M. In the one-way ANOVA, followed by Dunnet’s test and compared with the control group, p<0.05 was considered significant.


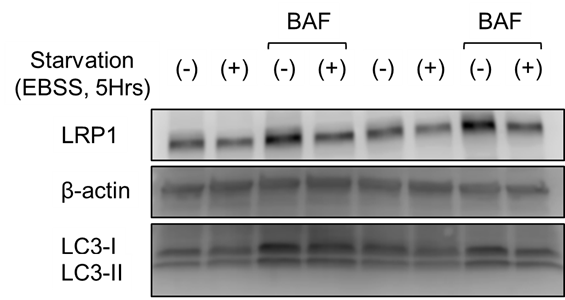


**Supplementary Figure 3.** BAF prevents LRP1 protein degradation in starved HepG2 cells. Autophagy was induced by starvation with EBSS for 5 h. LRP1 protein levels were then analyzed by Western blot. The results demonstrated that BAF prevented LRP1 degradation (starved BAF-treated cells vs starved control cells). LC3 isoforms are displayed in this figure as a control for autophagy induction.


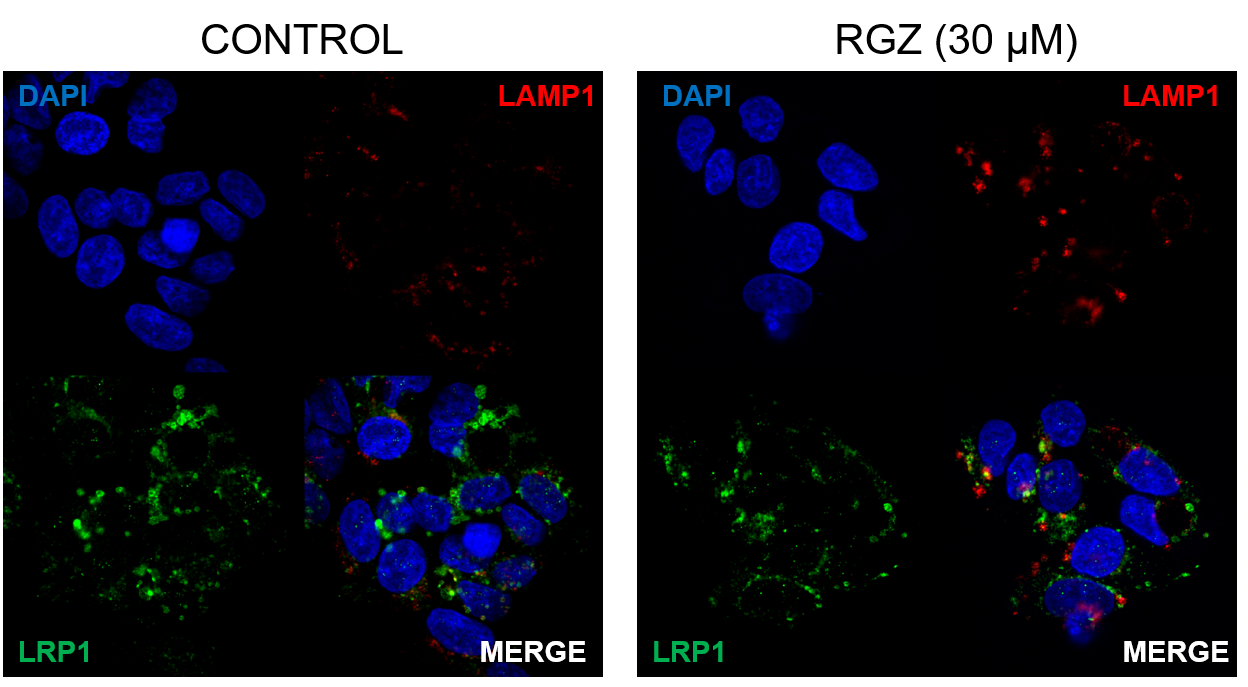


**Supplementary Figure 4.** 2D pictures from the 3D-reconstruction confocal microscopy.
